# Hypoxic Extracellular Matrix Preserves Its Competence after Expansion of Human MSCs under Physiological Hypoxia In Vitro

**DOI:** 10.3390/biomimetics8060476

**Published:** 2023-10-07

**Authors:** Diana Matveeva, Sergey Buravkov, Elena Andreeva, Ludmila Buravkova

**Affiliations:** Institute of Biomedical Problems of Russian Academy of Sciences, Moscow 123007, Russia; matveeva.dajana@yandex.ru (D.M.); sergey@wolf.ru (S.B.); buravkova@imbp.ru (L.B.)

**Keywords:** multipotent mesenchymal stromal cells, cell-derived matrix, physiological hypoxia, osteo-commitment

## Abstract

Tissue-relevant O_2_ levels are considered as an important tool for the preconditioning of multipotent mesenchymal stromal cells (MSCs) for regenerative medicine needs. The present study investigated the quality and functions of the extracellular matrix (ECM) of MSCs under low O_2_ levels. Human adipose tissue-derived MSCs were continuously expanded under normoxia (20% O_2_, N) or “physiological” hypoxia (5% O_2_, Hyp). Decellularized ECM (dcECM) was prepared. The structure of the dcECM was analyzed using confocal laser and scanning electron microscopy. Collagen, dcECM-N, and dcECM-Hyp were recellularized with MSC-N and further cultured at normoxia. The efficacy of adhesion, spreading, growth, osteogenic potential, and paracrine activity of recellularized MSC-N were evaluated. At low O_2_, the dcECM showed an increased alignment of fibrillar structures and provided accelerated spreading of MSC-N, indicating increased dcECM-Hyp stiffness. We described O_2_-dependent “ECM-education” of MSC-N when cultured on dcECM-Hyp. This was manifested as attenuated spontaneous osteo-commitment, increased susceptibility to osteo-induction, and a shift in the paracrine profile. It has been suggested that the ECM after physiological hypoxia is able to ensure the maintenance of a low-commitment state of MSCs. DcECM, which preserves the competence of the natural microenvironment of cells and is capable of “educating” others, appears to be a prospective tool for guiding cell modifications for cell therapy and tissue engineering.

## 1. Introduction

Multipotent mesenchymal stromal cells (MSCs) are considered to be one of the key populations involved in maintaining homeostasis in various tissues. These cells can be easily isolated from available tissue sources and expanded in vitro [1,2]. The resulting cell products have high secretory activity and are capable of trilineage stromal differentiation. Therefore, they are in demand for the needs of regenerative medicine and tissue engineering [3]. Currently, soluble secreted components of MSCs are positioned as a valuable cell therapy tool, comparable in efficacy to MSCs themselves, but safer [4]. It was convincingly demonstrated that a conditioned medium of MSCs was very effective in supporting local tissue resident cells, and in stimulating angiogenesis, neurogenesis, and immunomodulation [5,6]. A.I. Caplan has suggested the term “an injury drugstore” to describe MSC paracrine capacities [7]. Recently, the extracellular matrix (ECM) produced by MSCs has also attracted increasing interest. ECM components play an important role in maintaining the integrity of local tissue niches via their structural function and the deposition/release of growth factors and other mediators [8,9,10]. In addition, the products of limited proteolysis of ECM components have been shown to be highly biologically active. They regulate numerous biological processes such as autophagy, angiogenesis, adipogenesis, fibrosis, tumor growth, metastasis, and wound healing [9,11,12]. A number of peptides derived from collagen have been shown to possess anti-angiogenic and/or anti-tumor properties [13]. Fragments of both glycoproteins and elastin have been shown to enhance angiogenesis and to exhibit anti-angiogenic activity with greater efficiency than the full-length molecules [14]. It was convincingly demonstrated that a conditioned medium of MSCs was very effective in supporting local tissue resident cells, and in stimulating angiogenesis [9], neurogenesis [10], and immunomodulation [11]. 

Current cell culture technologies make it possible to obtain sufficient quantities of MSCs and MSC-derived products. In addition, ex vivo conditions provide a useful engine for MSC modification. So-called preconditioning or priming allows for the modulation or enhancement of specific MSC functions, such as angiogenic or migratory activities as examples [15,16]. Microenvironmental settings, such as levels of O_2_, glucose, growth factors, pH, and others, regulate the properties of MSCs and can be used for their preconditioning in vitro [17]. 

Short-term hypoxic preconditioning which mimics acute hypoxic stress or anoxia (0.1–1% O_2_) is widely used to enhance the angiogenic and migratory potentials of MSCs [18]. Meanwhile, a number of studies have shown that permanent low O_2_ levels are better suited to tissue physiological conditions, since, for many cell types, 1–5% O_2_ levels are not considered as hypoxia but in situ normoxia or so-called physiological hypoxia [19]. In some tissues, as in endosteal areas in bone marrow where hematopoietic stem cells and osteoblasts are localized, the O_2_ concentration is about 0–0.5% [20,21], whereas in the perivascular niche where MSCs and committed hematopoietic progeny are located, it is 3–5% [22,23]. In the perivascular MSC niche, the O_2_ concentration is higher, e.g., in adipose tissue it is 2–8% O_2_ [24].

Tissue-related physiological hypoxia in the microenvironment of MSCs is preferable for maintaining their low-committed state [25,26].

Previously, we demonstrated that permanent expansion under physiological hypoxia ensured significant modification of MSC functions manifested in increased proliferative potential, attenuated response to differentiation stimuli, and increased angiogenic activity [25,27]. These changes appeared to be related to the increased contribution of glycolysis to ATP production [28]. MSCs produced a well-developed ECM at both physiological hypoxia (5% O_2_) and normoxia (20% O_2)_, with similar levels of collagenous and non-collagenous components but with differences in appearance [29]. Based on these observations, we supposed that ECM turnover and structure may also have been affected. The data concerning the effect of O_2_ deprivation on the ECM are mainly related to short-term hypoxic stress. It was demonstrated that ECM metabolism alteration was regulated via a HIF-1-dependent mechanism [30,31]. Both structural molecules of the ECM and enzymes involved in its remodeling have been shown to be transcriptional targets of this factor [32]. The post-translational modification of collagens and its extracellular remodeling via the HIF-1-dependent mechanism have been demonstrated to play important roles in this process [33]. These observations provided the initial premise for our hypothesis that the properties of the ECM synthesized by MSCs under continuous hypoxia would change, persist, and influence cell behavior after subsequent recellularization. 

Recently, we demonstrated that MSCs produced a well-developed ECM at both physiological hypoxia (5% O_2_) and normoxia (20% O_2)_, with similar levels of collagenous and non-collagenous components but with differences in appearance [29]. We hypothesized that the ECM derived from MSCs under physiological hypoxia could retain its capacity after decellularization and maintain hypoxia-specific effects, which may be required for the targeted modification of MSC functions. We referred to this putative ability of the ECM as hypoxic competence. 

In the present work, we obtained and characterized the ECM derived from MSCs during continuous expansion under normoxic (20% O_2_, N) or “physiological” hypoxic (5% O_2_, Hyp) conditions. We were able to show that hypoxia-derived ECM is able to maintain “hypoxic” competence. After recellularization, MSCs on dcECM-Hyp demonstrated attenuated osteo-commitment, but were more susceptible to osteo-induction. These observations support the importance of the ECM in supporting the low commitment phenotype of MSCs. 

## 2. Materials and Methods

### 2.1. Cell Culture

MSCs from the stromal-vascular fraction of human adipose tissue were obtained from the Cell Physiology Laboratory cryo-collection. The experimental protocols were approved by the IBMP RAS Commission on Medical Bioethics (550/MSK dated 22 July 2020). After thawing, MSCs were cultured in Petri dishes with minimum essential media alpha (α-MEM) (Gibco, Thermo Fisher Scientific, Horsham, UK) supplemented with penicillin/streptomycin (100 units/mL and 100 mg/mL, respectively (PanEco, Moscow, Russia)) and fetal bovine serum (10% vol/vol, (HyClone Laboratories Inc., South Logan, UT, USA)). For normoxia (20% O_2_, N), MSCs were cultured in a standard CO_2_ incubator (5%CO_2_/95% air; 37 °C), and for “physiological” hypoxia (5% O_2_, Hyp) in a multi-gas incubator at 5% O_2_ (5%CO_2_/residual N_2_; 37 °C) (both devices Sanyo, Tokyo, Japan). The seeding density was 3–3.5 × 10^3^ cells/cm^2^. The medium was changed every 3–4 days. At 70–80% confluence, cells were enzymatically detached using 0.25% trypsin-EDTA (Gibco, ThermoFisher Scientific, Horsham, UK) and passaged. In the experiments, the samples from 3–6 individual donors were used. MSCs from different donors were routinely validated and satisfied to minimal ISTC criteria [34]. The MSCs from passages 3–5 were used for all assays and all experiments were performed in triplicate. 

**Figure 1:**
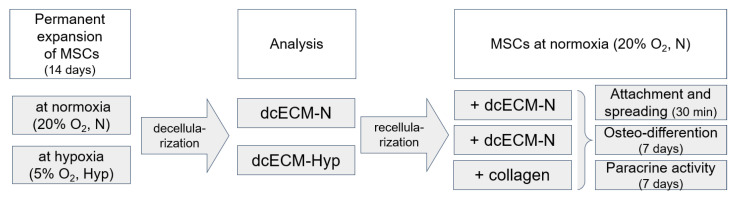
Study design. MSCs were permanently cultured under normoxia (20% O_2_, N) or “physiological” hypoxia (5% O_2_, Hyp) for 14 days. Decellularized ECMs (dcECMs) were examined using phase contrast microscopy, confocal laser scanning microscopy (LSM), and scanning electron microscopy (SEM). MSC-N were seeded on collagen coating, dcECM-N, and dcECM-Hyp and further cultured under normoxia and analyzed according to the experimental design.

The experimental design is presented in Figure 1. MSCs were continuously expanded under normoxia (20% O_2_, N) or “physiological” hypoxia (5% O_2_, Hyp). After 14 days, decellularized ECM (dcECM) was prepared. The structure of the dcECM was examined using phase-contrast microscopy, confocal laser microscopy (LSM), and scanning electron microscopy (SEM). The collagen coating, dcECM-N, and dcECM-Hyp were recellularized with MSC-N and further cultured at normoxia (20% O_2_). The efficacy of adhesion, spreading, growth, osteogenic potential, and paracrine activity of recellularized MSC-N were investigated. 

### 2.2. Preparation of Decellularized Matrices

The samples of dcECM were prepared as previously described [35]. Briefly, MSCs were seeded on culture plates or coverslips at a density of 5 × 10^3^/cm^2^ and cultured for 14 days. When the cells reached 100% confluence, 2-phospho-L-ascorbate sodium (50 μg/mL) (Fluka, Merck, Darmstadt, Germany) was added to stimulate the production of matrix components. 

MSCs were washed thrice with PBS (PanEco, Moscow, Russia) and treated with 0.5% Triton-X100 + 20 mM NH_4_OH (both Merck, Darmstadt, Germany) in PBS at 37 °C for 5 min. Cell lysis was monitored using phase-contrast microscopy. After incubation, cell debris was removed using PBS. To exclude DNA contamination, the preparations were treated with type I DNAse 50 U/mL (SciStore, Moscow, Russia) for 30 min at 37 °C.

The ECM after decellularization was designated as dcECM-N or dcECM-Hyp for MSC cultures at 20% or 5% O*_2_*, respectively. 

#### Analysis of the Decellularized Matrices 

Sirius Red F3BA dye (0.1%) (Sigma, Burlington, MI, USA) was used to assess collagenous protein deposition in the dcECM. 

The MSC-derived ECM structures were analyzed using SEM. Samples were washed twice with PBS and fixed overnight in a mixture of 2.5% glutaraldehyde and 2% paraformaldehyde (both Merck, Darmstadt, Germany) in PBS. Specimens were processed and dehydrated using increasing grades of ethanol (25%, 50%, 75%, 90%, 100%) for 15 min at each step. Dehydrated samples were dried using a critical point dryer and sputtered with gold. Samples were examined using a scanning electron microscope (CAMSCAN, Tokyo, Japan). 

DcECM specimens were fixed in 4% paraformaldehyde (Merck, Darmstadt, Germany) and examined using an LSM-780 confocal microscope (Carl Zeiss, Oberkochen, Germany). The autofluorescence of collagen fibers was detected by exciting the samples with a 488 nm argon gas laser, with a green color emission according to [36,37]. 

Measurements of the fibers’ orientations were performed using OrientationJ plugins in ImageJ software (National Institutes of Health, Bethesda, MD, USA). Grayscale LSM images of dcECM fibers were analyzed and colorized with Hue, Saturation, and Brightness (HSB) maps such that the pixel hue corresponded to the angle of the local fiber orientation, which ranged from −90° to +90° relative to horizontal. On each SEM image, 6 random regions of interest (ROIs) (a total of 36 ROIs for each group) were selected for coherence analysis.

### 2.3. Recellularization of MSCs on Decellularized Matrices

Permanently cultured normoxic MSCs (MSC-N) were enzymatically detached and were seeded into 35 mm Petri dishes with dcECM (dcECM-N or dcECM-Hyp). For recellularization experiments, rat collagen type I-coated plates (10 µg protein/cm^2^ (IMTEK, Moscow, Russia)) were considered as the control ECM (collagen). 

#### 2.3.1. Morphology and Actin Cytoskeleton of MSCs

For the analysis of adhesion and spreading, MSCs were seeded on dcECM-N, dcECM-Hyp, or collagen at a density of 3.5 × 10^3^/cm^2^. After 30 min, non-adherent cells were removed, and the adherent MSCs were fixed with 4% paraformaldehyde to calculate the efficiency of attachment and to evaluate the cell morphology and cytoskeletal structures. The actin cytoskeleton was detected using Alexa488-labelled phalloidin according to the manufacturer’s instructions (Invitrogen, Thermo Fisher Scientific, Waltham, MA, USA). Cells were analyzed using an LSM-780 confocal microscope (Carl Zeiss, Oberkochen, Germany).

The MSCs were categorized as follows after being attached: (1) small-sized unflattened cells (up to 50 µm); (2) medium-sized disc-shaped or elongated cells with F-actin in subplasmalemmal spaces (as a ring or in a leading edge) (50–100 µm); (3) large-sized flattened cells with pseudopodia F-actin presented in fibers and globules (100–200 µm). The total number of MSCs and number of MSCs with different morphologies were calculated in 10 randomly selected fields. The adhesion efficiency was expressed as the number of adherent cells per 1 mm^2^ of surface area. The heterogeneity of the MSCs was expressed as the percentage of MSCs with a different morphology.

#### 2.3.2. Paracrine Activity of MSCs 

To characterize the paracrine activity, the samples of conditioned medium were collected from MSCs cultured on different substrates for 7 days. The chemokines in the conditioned medium were measured using multiplex immunofluorescence analysis (MILLIPLEX^®^ MAP system, Merck Millipore, Darmstadt, Germany) according to the manufacturer’s instructions using the Human Cytokine/Chemokine Panel (41-plex). Samples were analyzed on a MAGPIX (Luminex, Chicago, IL, USA). Concentrations were automatically calculated using xPONENT 4.2 software (Luminex, Chicago, IL, USA).

### 2.4. Osteogenic Potential of MSCs

Spontaneous osteo-commitment and induced osteo-differentiation were characterized. For osteo-induction, dexamethasone (0.01 μM), β-glycerophosphate (10 mM), and L-ascorbic acid phosphate (0.2 mM) (all Merck, Darmstadt, Germany) were added to the growth medium. 

After 7 days, recellularized MSC-N was fixed with 4% paraformaldehyde and the activity of alkaline phosphatase—an early marker of osteo-differentiation—was detected using an Alkaline Phosphatase Kit (Sigma-Aldrich, Merck, St. Louis, MI, USA) according to the manufacturer’s instructions. After staining, the specimens were washed with distilled water and dried. For semi-quantitative analysis, the insoluble product of the histochemical reaction was extracted using dimethyl sulfoxide (DMSO (PanEco, Moscow, Russia)) and the staining intensity was measured using a spectrophotometer at λ = 550 nm (Biorad, Hercules, CA, USA).

For quantitative real-time RT-PCR of osteo-marker genes (*RUNX2*, *ALPL*, *COL1A1*), total RNA was extracted from MSCs using QIAzol lysis reagent (Qiagen, Germantown, MD, USA) using phenol-chloroform extraction, followed by DNAse treatment. Reverse transcription was performed using a QuantiTect Reverse Transcription Kit (Qiagen, CA, USA). The concentration and purity of RNA and cDNA in the samples were monitored using a Nanodrop ND-2000c spectrophotometer (Thermo Fisher Scientific, Waltham, MA, USA). The cDNA obtained was used for quantitative PCR in the presence of SYBR Green 1 (Syntol, Moscow, Russia). The cDNA was mixed in PCR tube strips (Eppendorf, Hamburg, Germany) with RT2 SYBR Green/ROX PCR master mix (Qiagen, Germantown, MD, USA) and the corresponding predesigned primers (qPCR Primer Assay, Qiagen, Germantown, MD, USA) for expression analysis of the following genes for which the reference mRNA sequence number is noted in parentheses: RUNX2 (NM_004348), ALPL (NM_000478), COL1A1 (NM_000088), HPRT1 (NM_000194), RPLP0 (NM_001002). All gene expression levels were determined using the 2^−ΔΔCt^ method, and normalized to the *HPRT1* and *RPLP0* housekeeping genes.

### 2.5. Microscopy 

Bright-field and phase-contrast microscopy was performed using a Nikon Eclipse Ti-U microscope equipped with a DS-Ri1 color digital camera. Images were stored and later processed using NIS-Elements Auto Research software (Nikon Instruments, Tokyo, Japan). SEM was performed using a scanning electron microscope (CAMSCAN, Tokyo, Japan). For LSM, a confocal microscope LSM-780 (Carl Zeiss, Oberkochen, Germany) was used. 

### 2.6. Statistical Analysis

Statistical analysis was performed using GraphPad software. All experiments were performed in at least three independent settings. The data are presented as mean ± standard error of the mean (M + S.E.M.). The statistical reliability of differences between the two data groups was assessed using the non-parametric Mann–Whitney test and Wilcoxon t-criteria for small sample groups at the selected significance level of *p* < 0.05.

## 3. Results and Discussion

Tissue-related O_2_ levels during permanent ex vivo expansion of MSCs are assumed to adequately mimic the native microenvironment of these cells and are considered as “physiological” hypoxia. The significant alterations of MSC functions under low O_2_ have been described elsewhere [16,18,26]. MSCs demonstrated increased proliferation, and attenuated osteo-, adipo-, and enhanced chondrogenic potentials [17,36]. The alteration in cellular metabolism supported these changes in MSC functions. Previously, we showed that MSCs, when permanently expanded under 1–5% O_2_, had lower cellular ATP levels than MSCs in 20% O_2_, attenuated mitochondrial ATP production, and increased contribution of glycolysis in ATP production [28].

### 3.1. Decellularized Extracellular Matrices

The data cited above led to the assumption that the structure and functions of the ECM under “physiological” hypoxia might differ from those under ambient O_2_. Indeed, as we recently demonstrated, in vitro MSCs produced a well-developed ECM under both “physiological” hypoxia and normoxia. In the spaces between MSCs, the ECM-N appeared as a dense structure with no clear separation between individual fibrils, whereas the ECM-Hyp consisted of a network of thin intersecting fibrils [29].

**Figure 2:**
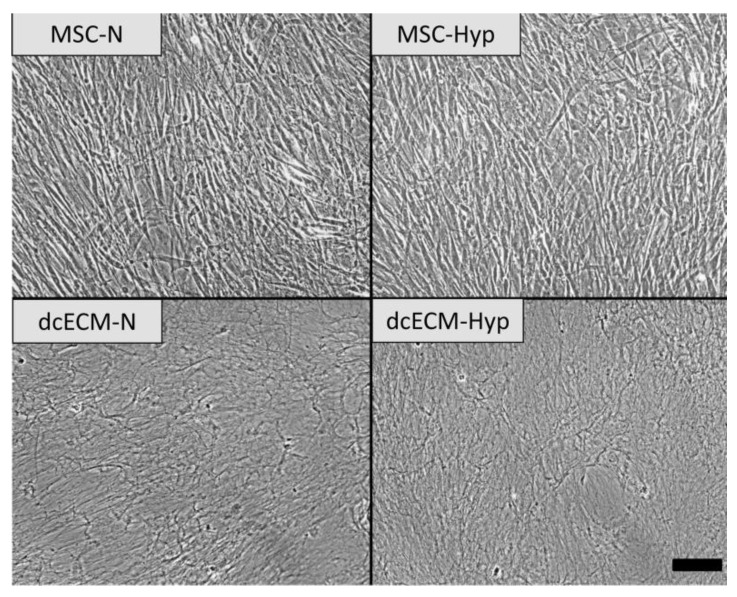
MSC monolayers before (**upper** row) and after (**lower** row) treatment with decellularization solution. Representative phase-contrast images. Scale bar—100 um.

In the present work, we used the same protocols for MSC continous expansion at normoxia or “physiological” hypoxia. Dense monolayers of MSCs (Figure 2) were decellularized. The efficacy of decellularization was monitored using DAPI staining. The decellularized ECM (hereafter referred to as dcECM-N or dcECM-Hyp) was represented by a well-developed network (Figure 2). The morphology and guidance potential were examined. 

#### Morphology of Decellularized Matrices

**Figure 3:**
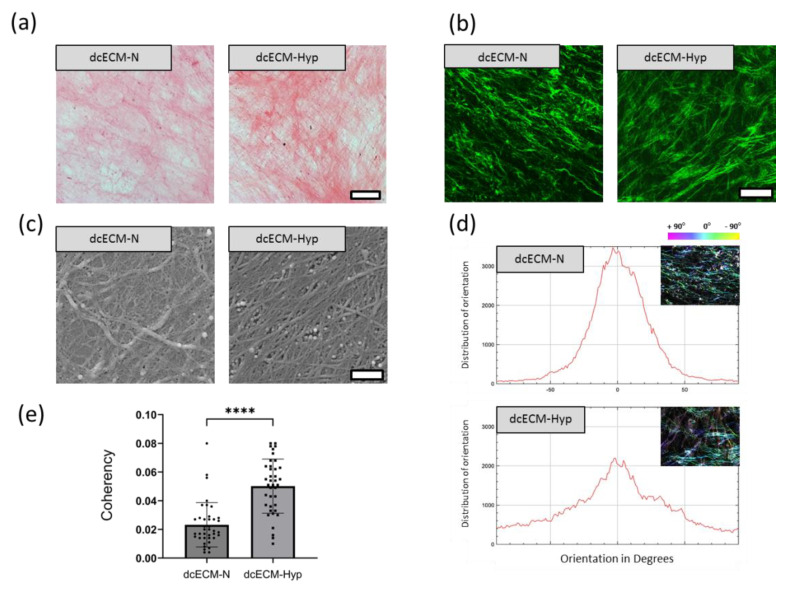
Characterization of decellularized extracellular matrices of MSCs cultured at 20% (dcECM-N) or 5 % O_2_ (dcECM-Hyp). Representative images: (**a**) collagenous proteins stained with Sirius Red, bright field, scale bar—50 μm; (**b**) laser-scanning confocal microscopy (LSM), autofluorescence of dcECM at 488 nm, scale bar—50 μm; (**c**) scanning electron microscopy (SEM), scale bar—500 nm. Coherency image analysis (performed using ImageJ): (**d**) fibril direction distribution, inserts—Hue, Saturation, and Brightness (HSB) maps generated using the OrientationJ plugin; (**e**) coherency analysis of different dcECMs. Data are presented as mean ± SD, **** *p* < 0.0001 (*n* = 6).

Bright-field, confocal, and scanning electron microscopy approaches were used to examine the dcECM networks. Sirius Red F3BA, a histological dye that binds specifically to fibrillar collagenous proteins, revealed a persistence of ECM structures after decellularization (Figure 3a). ECM structures are known to autofluoresce at a wavelength of 488 nm, so we used confocal microscopy to detect unstained ECM fibrils. Differences in packing patterns were observed: in dcECM-N, a dense network of tortuous fibrils was observed, while in dcECM-Hyp, aligned fibrils were arranged in alternating dense and sparse areas (Figure 3a,b). SEM was used to confirm these observations. As SEM provides resolution down to the nanometer scale, we were able to observe membrane-like structures of different-sized crossed tortuous fibrils in dcECM-N. The dcECM-N was presented as uniformly aligned coaxial fibrils assembled into grid-like structures (Figure 3c,d).

The angle measurements for dcECM-N and dcECM-Hyp show a random distribution (0° ± 90°), where 0° is the dominant horizontal direction. The inserts in Figure 3d show HSB maps generated using the OrientationJ plugin. At the same time, dcECMs show a more aligned distribution (0° ± 50°) while the fibers in dcECM-Hyp have multidirectional angular deviations from the dominant direction. We found a significant increase in the coherency of dcECM-Hyp fibers (Figure 3e).

The different patterns of dcECM morphology were similar to the appearance of ECM in the spaces between MSCs cultured under 20% or 5% O_2_, regarding which we recently published [29]. This concordance confirmed the adequate preservation of ECM structures after decellularization. The differences in density and alignment between ECMs under different O_2_ levels are probably determined by the qualitative composition of the ECM, including both structural and regulatory components. It has been shown that the extracellular modification of fibrillar collagens is hypoxia-dependent via increased production of lysyloxidases (LOX) and lysyloxidase-like enzymes (LOXL-2 and LOXL-4), which provide cross-links between collagen fibers [37,38]. These aligned, tightly packed fibrils promote cell migration. Makris et al. showed that expansion of chondrocytes at low O_2_ (4%) over 3–4 weeks significantly increased LOX expression and was associated with an increase in cross-links between collagen fibrils and increased stiffness of the ECM [39]. 

Cell–ECM interaction is bidirectional. Physical forces transmitted through the ECM can directly regulate the activity of nuclear transcriptional regulators, thereby directing cell fate [40]. The ECM-dependent spatial-mechanical regulation of cell phenotype, such as determination of cell shape, cell migration activity, and differentiation potential, has been demonstrated [41].

Based on our observations, we supposed that ECM-Hyp would appear different from ECM-N not only morphologically but also functionally, resulting in divergence of ECM activity. The preservation of structural and topological features determined by physiological hypoxia after decellularization seems promising for the use of cell-derived dcECM as a modulator of cellular behavior.

### 3.2. Recellularization of Decellularized Matrices

To characterize the effects of O_2_ levels on the functional properties of the ECM, dcECMs were prepared from MSCs at 20% or 5 % O_2_. Collagen type I coating was used as a reference. In recellularization experiments, MSCs that were continuously cultured at 20% O_2_ were used. An MSC suspension (2 × 10^3^/cm^2^) was inoculated onto different substrates and further cultured at 20% O_2_ (MSC-N). 

#### 3.2.1. Attachment and Spreading of MSCs on Decellularized Matrices 

MSCs are known to adhere rapidly onto plastic culture ware. As shown by [42], 30 min was sufficient for MSC attachment and the initiation of spreading. We used this time point to compare the efficacy and patterns of these events on different substrates. The F-actin cytoskeleton was stained with Alexa488-phalloidin. 

**Figure 4:**
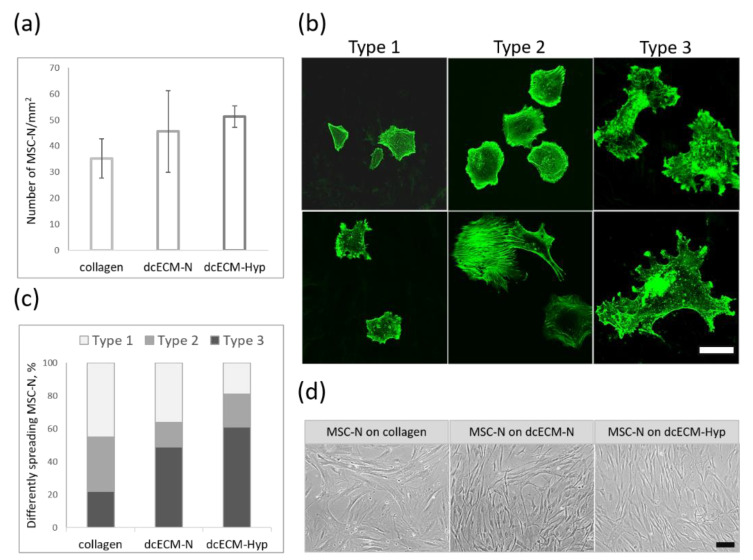
The attachment, spread, and growth of MSCs on different substrates: (**a**) Number of attached MSCs on collagen/dcECM-N/dcECM-Hyp; (**b**) Heterogeneity of MSC spreading: Type 1—small-sized unflattened cells, Type 2—medium-sized disc-shaped or elongated cells with F-actin in subplasmalemmal spaces; Type 3—large-sized flattened cells with pseudopodia, F-actin presented in fibers and globules. Representative images. Scale bar—100 um. LSM, Alexa488-phalloidin; (**c**) The proportion of differently spreading MSCs after 30 min of adhesion on collagen/dcECM-N/dcECM-Hyp; (**d**) Culture of MSC-N, 72 h after recellularization on collagen/dcECM-N/dcECM-Hyp. Representative images, phase-contrast, scale bar—100 um.

We did not find a significant difference in the number of attached MSCs on different dcECMs or collagen, although attachment seemed to be more effective on dcECM-Hyp (Figure 4a). MSCs showed heterogeneity in size and morphology. Following [43,44], we distinguished the following morphological types (Figure 4b): (1) small-sized unflattened cells; (2) medium-sized disc-shaped or elongated cells with F-actin in subplasmalemmal spaces (as a ring or in a leading edge); (3) large-sized flattened cells with pseudopodia. The MSC population on collagen was enriched in small-sized MSCs, whereas the large-sized flattened-cell MSCs were abundant on dcECM. The proportion of such MSCs was significantly higher among dcECM-Hyp-attached (Figure 4c). These observations can be interpreted as follows. On the one hand, it has previously been well demonstrated that fibroblasts undergo the consecutive steps of cell shape changes during spreading and movement during the first 2–24 h after attachment onto plastic ware [43,44,45], namely: disc-shaped, polarized with active edge, elongated fibroblast-like. Thus, the different shapes of MSCs at the same time point may correspond to the intensity of spreading and the potential for movement. Our data suggest that dcECMs provided a more favorable surface for spreading, as the proportion of disc-shaped, elongated, and flattened cells with pseudopodia was higher than that in collagen. It could be argued that the physical and topographical properties of dcECMs may have shortened the attachment time for MSCs, allowing them to become discoid/elongated and then flattened more quickly. These suggestions were supported by the decrease in the proportion of small non-spreading forms in favor of flattened forms, especially on dcECM-Hyp (Figure 4c). 

On the other hand, we observed marked differences between normoxic and hypoxic dcECMs, suggesting differences in their quality. Matrix stiffness is known to influence cell size and morphology [1,2,3]. On soft substrates, cells spread to a lesser extent and they are rounder than on stiff substrates. On stiff substrates, the same cells spread to a greater extent. The F-actin in such cells is organized in concentric structures under the cell membrane or bundles of stress fibers [46]. In line with these findings, we suppose that our data may be considered as an indirect indication that dcECM-Hyp can be stiffer than dcECM-N, allowing for the more rapid spread of MSCs. Our observations of the more aligned fibrils of dcECM-Hyp (Figure 2) may also be an indication of this suggestion. 

After 3 days of culture, the MSCs looked quite similar on both dcECMs (Figure 4d). The cells had a particular fibroblast-like appearance with a narrow, elongated body and a few cell processes. The MSCs on collagen were markedly different, being flattened and larger in size (Figure 4c). 

#### 3.2.2. Paracrine Activity of MSCs on Decellularized Matrices

In vitro, MSCs produce a wide range of bioactive molecules that are in high demand for use in regenerative medicine protocols. In addition, soluble mediators are now considered more attractive than cellular products. They have fewer limitations in clinical applications. [47]. 

The potential of the ECM or its components for stimulating paracrine activity has been demonstrated in several publications. For example, Peng et al. showed that the expansion of MSCs on laminin was accompanied by an increase in GRO-α, HGF, and IL-8. As they demonstrated a paracrine role for these cytokines in reducing cardiomyocyte apoptosis in experimental myocardial ischemia, they suggested that the laminin coatings could be used to prime MSCs for cell therapy needs [48]. The stimulatory effect of dcECM on cytokine production was demonstrated when MSCs and hematopoietic stem/progenitor cells (HSPCs) repopulated on the dcECM prepared from bone marrow MSCs. The cytokines involved in intercellular signaling in the bone marrow niche, such as angiopoietin-1, SDF-1, IL-8, VEGF, and HGF, were elevated in comparison with MSC/HSPC co-cultures on standard culture plastic [49]. The conditioned medium of macrophages on MSC-derived ECM contained elevated levels of BMP2, FGF2, and TGFβ3 and stimulated the osteogenic differentiation of MSCs [50].

**Figure 5:**
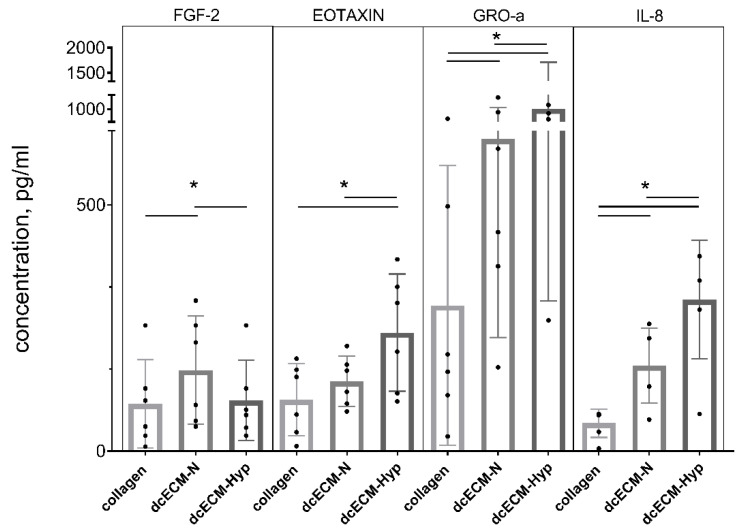
Paracrine activity of MSCs recellularized on collagen/dcECM-N/dcECM-Hyp: The data are presented as Median ± IQR, * *p* < 0.05 (*n* = 6).

In this paper, immunofluorescence multiplex analysis was used to assess 48 cytokines and growth factors in the conditioned medium of MSCs recellularized on collagen, dcECM-N, or dcECM-Hyp, and expanded for 7 days (Table S1 in Supplement). Some of them were poorly represented—the mediators were either not detected or detected at the sensitivity threshold of the method. The levels of IL-6, MCP-1, IP-10, and RANTES, were independent of the type of substrate (Figure 5. The decrease in FGF-2 and the increase in IL-8, GRO-α, and eotaxin levels were detected in the conditioned medium of MSCs recellularized on dcECM-Hyp compared to dcECM-N (Figure 5). As CXCL chemokines, GRO-α and IL-8 are strongly involved in cell migration and angiogenesis [51]. We noted the down-regulation of the pleiotropic growth factor FGF-2. This was quite unexpected as according to [52,53], it was expected to act in a unidirectional manner with GRO-α and IL-8. Meanwhile, these results could be interpreted based on the observations of Kim and Ma [54]. They showed that the ECM derived from MSCs under normoxia or hypoxia had different binding capacities for cell-secreted and exogenous FGF-2. DcECM-Hyp deposited twice as much FGF-2 than dcECM-N and bound more strongly to FGF-2 from the conditioned medium of MSCs and exogenous FGF-2. It is likely that a similar mechanism could occur in our case, reducing the soluble FGF-2 level in favor of the bound one.

Overall, the described changes in the paracrine profiles of dcECM-Hyp vs. dcECM-N may be involved in the phenomenon of the preservation of the “hypoxic” competence of dcECM-Hyp described below.

#### 3.2.3. The Effect of Decellularized Matrices on the Osteogenic Potential of MSCs

The potential for differentiating into osteogenic, adipogenic, and chondrogenic lineages is considered to be the most important capacity of MSCs [2,55]. It has been well demonstrated that the efficacy of in vitro differentiation is strongly dependent on O_2_ levels in the microenvironment. According to numerous observations, osteo- and adipo- differentiation are attenuated, while the chondrogenic one is accelerated [16,28,36]. In particular, induced osteo-differentiation of MSCs under “physiological” hypoxia is reduced compared to that under ambient O_2_, both at the transcriptional level and in the production of specific osteo-markers [28,56]. 

**Figure 6:**
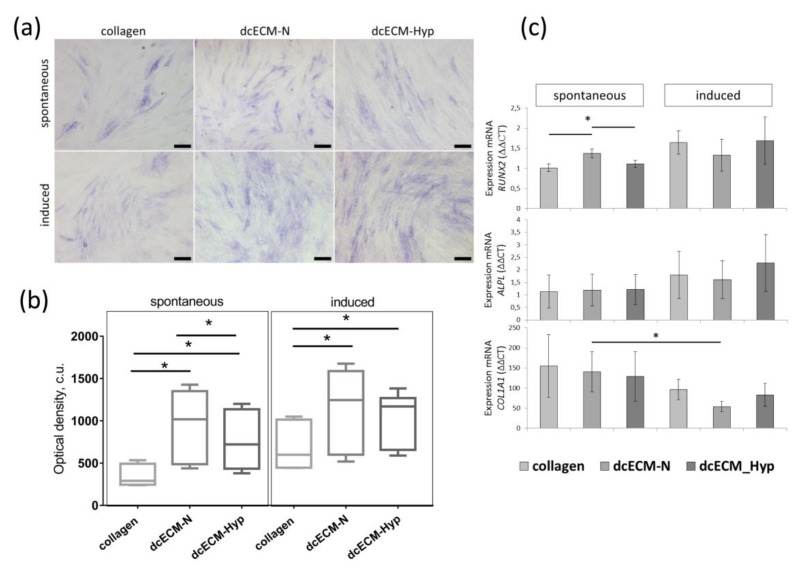
Osteogenic potential of MSC-N after recellularization on collagen/dcECM-N/dcECM-Hyp (spontaneous osteo-commitment and induced osteo-differentiation): (**a**) Histochemical detection of alkaline phosphatase (ALP) activity. Representative bright-field images, Scale bar—100 um; (**b**) Colorimetric determination of ALP staining intensity. Data are presented as Median ± IQR, * *p* < 0.05, (*n* = 6); (**c**) The relative expression of osteogenesis-related genes (*RUNX2*, *ALPL*, *COL1A1*). Data are presented as mean ± SD, * *p* < 0.05, (*n* = 3).

To verify our hypothesis that the dcECM-Hyp is able to preserve the hypoxic competence, we compared the efficacy of spontaneous osteo-commitment and induced osteo-differentiation of MSCs on dcECM-Hyp and dcECM-N. After recellularization on collagen or both dcECMs, MSC-N were cultured in a standard CO_2_ incubator under ambient O_2_ (20%, MSC-N). The signs of spontaneous commitment and the response to osteo-induction were assessed (Figure 6). 

After 7 days, the activity of alkaline phosphatase (ALP)—an early marker of osteo-differentiation—was assessed histochemically (Figure 6a). The MSC population was heterogeneous with respect to ALP activity. Cells with high, medium, and low levels of staining were detected. At the same time, heterogeneity was more pronounced among MSCs without osteo-induction. MSCs with high ALP activity on collagen were represented by large-sized cells, while there were both large and small spindle-shaped positively stained cells present on dcECM. Osteo-induction resulted in an increase in ALP-positive MSCs on all dcECMs. On collagen, stained MSCs formed small clusters, whereas ALP-positive MSCs were more evenly distributed on dcECM (Figure 6a). 

Semi-quantitative colorimetric analysis revealed that, in spontaneously osteo-committed MSCs, the intensity of ALP-staining was significantly higher on both dcECMs vs. collagen. The intensity of MSC staining on dcECM-N was 1.5-fold higher than that of MSCs on dcECM-Hyp (*p* < 0.05) (Figure 6b). This effect was abolished after osteo-induction. The intensity of ALP staining was similar in both the dcECM-N and dcECM-Hyp groups (Figure 6b). 

Further, we examined the transcriptional activity of osteogenesis-related genes: transcription factor *RUNX2*—master gene of osteo-differentiation; *ALPL*—gene encoding ALP; and *COL1A1*—a major protein in the ECM of osteodifferentiated MSCs (Figure 6c). In the absence of osteo-inductors, *RUNX2* was up-regulated in dcECM-N-expanded MSCs vs. collagen and dcECM-Hyp. The transcript levels of *ALPL* and *COL1A1* were not different in all groups (Figure 6c). After osteo-induction, only a trend towards up-regulation of *ALPL* and *RUNX2* was observed. Meanwhile, the increase was more pronounced on dcECM-Hyp vs. dcECM-N (Figure 6c).

On the contrary, *COL1A1* was down-regulated upon osteo-induction. A significant decrease in *COL1A1* expression was found in MSCs on dcECM-N. Previously, a similar down-regulation of *COL1A1* in recellularized fibroblasts upon osteo-induction was described by Nowwarote et al. [57]. It could be suggested that collagen as a single substrate or in the ECM performs a negative feedback loop, controlling collagen production. 

The use of dcECM allows for the reproduction of a local microenvironment for MSCs similar to the native one, preserving the topology and deposited signals necessary for cell functions [58]. On dcECM, MSCs demonstrated accelerated migration [59], reduced intracellular levels of reactive oxygen species due to activation of the antioxidant system (superoxide dismutase and catalase) [60], enhanced colony-forming capacity, and differentiation [61,62].

A significant elevation in the MSC capacity for differentiation on dcECM compared to culture plastic ware has been confirmed in several studies [63,64]. Xiong et al. [65,66] demonstrated an increase in the expression of the key osteo-markers transcription factor *RUNX2* and osteocalcin, as well as increased mineralization of the osteo-matrix in mouse MSCs cultured on human MSC-derived dcECM [61]. The osteo-inductive properties of MSC-derived ECM may be manifested by a combination of proteins such as collagen 1, tenascin C, vitronectin, fibronectin, and bound growth factors (e.g., TGF-β, CTGF, CYR61) and morphogens (e.g., BMP-2) [63]. 

Our study revealed the phenomenon of the preservation of hypoxic competences of ECM produced by MSCs under physiological hypoxia. This was manifested as follows. When MSC-N were seeded on dcECM-Hyp and subsequently expanded under ambient O_2_ (N), MSC-N showed a decrease in osteo-potential. Previously, a similar effect had only been described by Pei et al. [16]. The authors used dcECM derived from MSCs preconditioned in hypoxia for 14 days. After subsequent recellularization with MSCs, a decrease in differentiation in the osteo- and adipo-directions was observed. 

A low (relative to ambient) level of O_2_ is one of the most important physical factors of the tissue microenvironment of MSCs [25]. Analysis of the effects of reduced O_2_ levels on the differentiation potential of MSCs from different tissue sources showed a decrease in osteo-differentiation both at the levels of transcription of osteo-genes and the expression of encoded matrix molecules. After osteo-induction of MSCs continuously cultured at tissue-relevant O_2_ (1–10%), osteo-genes were down-regulated: *SPP1* (osteopontin), *BGLAP* (osteocalcin)], *IBSP* (bone sialoprotein binding integrin), *COL1A1* (type I collagen), and the major osteogenic transcription factor *RUNX2* [64,67,68]. Short-term anoxia (~0.02% O_2,_ 12 h) also provoked attenuated osteo-induction vs. 20% O_2_. The effects were manifested as down-regulation of *RUNX2*, *COLI*, *COLIII*, *COLVI*, and *SPPI* as well as weak matrix mineralization [56,69]. 

In our study, the manifestations of the dcECM-Hyp effects on MSC osteo-potential were mainly related to spontaneous osteo-commitment. In the presence of osteo-inducers, there was an alignment of the osteo-differentiation manifestations. The mechanism explaining these changes has not been established. From our data, the differences in ECM packaging as well as in levels of chemokines could be considered. In terms of MSC osteo-potential, the change in the soluble mediators’ profile of MSCs on dcECM-Hyp is interesting. We detected an increase in the inflammatory cytokines GRO-a and eotaxin, which are considered as potential predictors of bone mass loss, i.e., may negatively affect osteogenesis. The anti-inflammatory dexamethasone is a constitutive component of the osteo-inductive medium, and a decrease in GRO-a has been demonstrated in MSCs following osteo-induction in vitro [70]. Thus, osteo-inducers may mask dcECM-Hyp tutoring. As for IL-8, this cytokine has been shown to promote chondrogenic differentiation [71]. Since chondro- and osteo-differentiation are reciprocally regulated, it can be suggested that increased levels of this inflammatory cytokine may inhibit osteo-commitment. The observed decrease in FGF-2 could be discussed as another reason for the attenuated osteo-potential of MSCs on dcECM-Hyp. FGF-2 is considered as one of the osteo-inductive cytokines whose receptor, FGFR1, is a key regulator of osteoblast maturation during osteogenesis. The reason for the decrease in FGF-2 in the conditioned medium remains to be elucidated. It may be related either to a decrease in the availability of the ECM-anchored FGF-2, as shown by Kim and Ma [54], or to the activity of the corresponding receptor. Therefore, at least one mechanistic insight into the realization of dcECM’s education program has been demonstrated—shifting the levels of paracrine mediators leading to decreased osteo-commitment. In addition, the qualitative data on the more aligned packaging of ECM fibrils are supported by the data from the spreading experiments that suggest an increase in dcECM-Hyp stiffness. The exact mechanisms of the “educating” capacity of dcECM are awaiting further elucidation. 

## 4. Conclusions

Our study confirmed that the structural and instructive properties of MSC-derived ECM are altered under physiological hypoxia. At low O_2_, we found an increased alignment of fibrillar structures, while MSC spreading during recellularization indirectly indicated an increase in the stiffness of hypoxic dcECM. Here, we described the phenomenon of an O_2_ level-dependent “ECM-educated” phenotype of MSCs. This was reflected in both the paracrine activity and osteo-commitment potential. It can be supposed that the ECM from physiological hypoxia is able to ensure the maintenance of the low-commitment state of MSCs. At the same time, the sensitivity of such MSCs to differentiation stimuli, in our case, to osteo-inducers, was increased. The proposed protocol for the production of dcECM, which preserves the competence of the MSC microenvironment and is capable of “educating” the other cells, appears to be a very promising tool for the targeted modification of cell properties for the purposes of cell therapy and tissue engineering.

## Data Availability

The data are available from the authors on personal request.

## Supplementary Materials

The following supporting information can be downloaded at: https://www.mdpi.com/article/10.3390/biomimetics8060476/s1, Table S1: Paracrine activity of MSCs recellularized on collagen/dcECM-N/dcECM-Hyp.
