# Supplementary material for: Hypoxic Extracellular Matrix Preserves Its Competence after Expansion of Human MSCs under Physiological Hypoxia In Vitro
Source: Biomimetics (Basel). 2023 Oct 7;8(6):476. doi: 10.3390/biomimetics8060476 (PMC10604705; doi:10.3390/biomimetics8060476)
Supplement: Supplementary file 1 [file biomimetics-08-00476-s001.zip › biomimetics-2498937-supplementary.pdf]

biomimetics

MDPI

Table S1. Paracrine activity of MSCs recellularized on collagen/dcECM-N/dcECM-Hyp

Cytokine	Collagen	DcECM-N	DcECM-Hyp
	Concentration, pg/ml		
EGF	5.86 ± 2.36	5.69 ± 2.20	5.23 ± 2.08
FGF-2	95.97 ± 89.67	164.47 ± 109.6	103.182 ± 81.47
Eotaxin	104.44 ± 73.42	141.65 ± 51	240.62 ± 118.87
TGF-A	ND	ND	ND
G-CSF	237.18 ± 330.33	461.9825 ± 530.96	300.49 ± 334.60
Fit3L	7.81 ± 2.086	7.76 ± 3.79	9.23 ± 2.68
GM-CSF	80.63 ± 9.34	291.275 ± 334.28	46.24 ± 51.85
Fractalkine	111.57 ± 81.66	164.59 ± 182.72	99 ± 51
IFNa2	20.24 ± 7.96	21.21 ± 10.26	24.29 ± 10.13
IFNy	ND	ND	ND
GRO	295.69 ± 283.87	634.2 ± 403.4	1009. 43 ± 704.48
IL-10	0	0	0
MCP-3	208.22 ± 203.79	227.63 ± 239.89	220.57 ± 185.67
IL-12p40	2.85 ± 2.04	3.27 ± 1.64	3.31 ± 1.98
MDC	19.14 ± 8.21	27.85 ± 16.81	27.88 ± 13.19
IL-12p70	ND	ND	ND
PDGF-AA	ND	ND	ND
IL-13	ND	ND	ND
PDGF-AB/BB	36.29 ± 6	41.54 ± 9. 22	38.42 ± 6Б91
IL-15	4.14 ± 1.31	4.57 ± 2.68	4.87 ± 2.51
sCD40L	18.83 ± 13.17	17.48 ± 2.68	14.71 ± 5.10
IL17-A	ND	ND	ND
IL-1RA	2.84 ± 1.59	5.25 ± 4.79	3.44 ± 1.77
IL-1A	ND	ND	ND
IL-9	ND	ND	ND
IL-1B	ND	ND	ND
IL-2	ND	ND	ND
IL-3	ND	ND	ND
IL-4	76.725 ± 69.17	82.18 ± 77.18	78.34 ± 61.55
IL-5	ND	ND	ND
IL-6	3602.75 ± 1693.58	4070 ± 856.21	4117.5 ± 1092.41
IL-7	7.43 ± 1.86	7.76 ± 1.83	8.07 ± 1.71
IL-8	56.91 ± 28.94	173.85 ± 76.1	307.9 ± 120.73
IP-10	4278.13 ± 7169.52	3075 ± 3476.06	3343.42 ± 4219.62
MCP-1	6568.25 ± 2084.44	6612.25 ± 2566.37	8435.75 ± 879.45
MIP-1a	11.61 ± 3.25	16.92 ± 16.84	12.93 ± 13.68
MIP-1b	ND	ND	ND
RANTES	1628.74 ± 2504.23	1344.18 ± 1500.75	1573.18 ± 1738.53
TNFa	ND	ND	ND
TNFb	ND	ND	ND
VEGF	ND	ND	ND

* Data are presented as mean±SD (n=6). ND (no detected) – cytokines, the level of which was higher (VEGF) or lower (others) than the sensitivity of the assay.
